# Association between reported ADHD symptom and motor development delay in preschool children

**DOI:** 10.3389/fped.2024.1480488

**Published:** 2024-11-22

**Authors:** Zhijun Cui, Shijie Li, Aimin Liang, Hongmei Huang, Xin Ni

**Affiliations:** ^1^ Department of Child Health Care Center, Beijing Children's Hospital, Capital Medical University, Beijing, China; ^2^ Department of Otolaryngology Head and Surgery, Beijing Children's Hospital, Capital Medical University, Beijing, China

**Keywords:** preschool children, attention-deficit/hyperactivity disorder (ADHD), fine motor, gross motor, developmental quotient

## Abstract

**Objective:**

To explore whether the motor developmental level is associated with the attention deficit/hyperactivity disorder (ADHD) symptoms severity reported by parents in preschool children.

**Methods:**

Preschool children aged 4–6 years old with the chief complaint of reported inattention or hyperactivity by kindergarten teachers or parents were recruited in this study. All participants were consulted by at least one experienced developmental behavior pediatrician, according to DSM-V diagnostic criteria of ADHD. Their neuromotor developments were assessed by the Children's Neuropsychological and Behavior Scale and recorded as developmental quotient (DQ) score in gross motor, fine motor, and other domains. Regarding the evaluation of ADHD symptoms, parents of the 4-year-old group completed the Conners' Parent Symptom Questionnaire, while parents of the 5-year-old group completed The Vanderbilt ADHD Diagnostic Parent Rating Scale.

**Results:**

A total of 137 preschool children aged 4–4.9 years (4-year-old group) and 252 were aged 5.0–5.9 years (5-year-old group) were included in the study. Children exhibiting ADHD symptoms were at a much higher risk of fine motor delays compared to gross motor delays, particularly among the younger age group. Correlation analysis and hierarchical regression showed that in the 4-year-old ADHD group, better gross motor development was associated with increased severity of parent-reported ADHD symptoms. In the 5-year-old ADHD group, poorer fine motor development was linked to higher ADHD symptom severity. For children who do not meet ADHD diagnostic criteria, no significant correlations were found between gross or fine motor developmental quotients (DQ) and the severity of ADHD symptoms.

**Conclusions:**

Preschool children exhibiting ADHD symptoms are at a notable high risk of fine motor delays. Motor development in preschool children who meet ADHD diagnostic criteria is related to the severity of their symptoms. It is important to monitor both fine and gross motor development in preschool children with ADHD.

## Introduction

Attention deficit/hyperactivity disorder (ADHD) is a neurodevelopmental disorder characterized by developmentally inappropriate levels of inattention, hyperactivity, and impulsiveness (1). ADHD symptoms often emerge in preschool children, with prevalence estimated about 5.5% (95% CI, 3.3–7.7) for preschoolers (2). Understanding the early characteristics of children with ADHD symptoms is of great significance for early identification and intervention.

Studies have noted motor skill delays in preschoolers with ADHD (3–5). However, the relationship between motor skills and ADHD symptoms remains unclear. Motor skills encompass gross and fine motor abilities, both significantly linked to executive function (6). Good gross motor skills were a risk factor for ADHD symptoms in infancy (7), and specific gross motor behaviors at early ages correlate with later ADHD severity (8, 9). Impaired fine motor skills were consistently observed in school-age children with ADHD (10, 11). However, little study research on the relationship between motor development in preschool children and ADHD symptoms.

Preschool years witness rapid sensorimotor brain area development (12), paralleling cognitive and motor skill maturation. Children with ADHD often exhibited atypical sensorimotor and cognitive brain network development (13). Increased ADHD severity was associated with altered white matter organization which support motor skills and cognitive abilities (14). It was also reported a strong correlation between motor skills and cognitive abilities (e.g., executive function) in typically developing preschool children (15, 16). Motor development is integral to academic, social, and communication skills, and delays or imbalances in motor development are common in neurological disorders like ADHD. The exact mechanisms undelaying ADHD remained unclear, but hypotheses suggested immature or dysfunctional brain connectivity involving motor areas and high order cognitive related brain areas.

Previous research noted motor delays in children with ADHD and their association with future symptom severity and brain development (14, 17). But the relationships between gross/fine motor development and ADHD symptoms in preschoolers remain unexplored. This study aimed to address this gap in children aged 4–6 years. Given the high activity levels of preschoolers, not all active children meet ADHD diagnostic criteria. This study included ADHD and ADHD symptom-like groups aged 4.0–5.9 years to examine the motor-symptom relationship. Hypothesis 1 proposed a delayed fine and gross motor development in preschool ADHD children. Hypothesis 2 proposed that symptom severity was associated with respective motor development levels. Exploring the motor-symptom association aids in understanding ADHD brain development mechanisms and differentiating symptom severity.

## Materials and methods

### Participants

All participants were recruited from the developmental behavior clinic in the same hospital between March 2021 and January 2024. The children met the following inclusion criteria were included: right-handed; aged from 4.0 to 5.9 years, with the chief complaint of inattention or hyperactivity reported by kindergarten teachers or parents. Participants were excluded for developmental quotient (DQ) below 70 on the Children Neuropsychological and Behavior Scale, language disorder, visual or hearing impairment, psychoactive medication use, and other neurological or psychiatric disorders except ADHD.

There were 137 children aged 4.0–4.9 years and 252 children aged 5.0–5.9 years, referred to as the 4-year-old group and the 5-year-old group, respectively. For each age group, children were categorized into either the ADHD group or the ADHD symptom-like group by one or two clinicians based on the DSM-5 criteria. During the diagnostic process, clinicians relied on at least two sources of information, such as interviews and observations conducted by the clinician, parent questionnaires, behavioral observations performed by trained professionals, and videos of the child's daily activities provided by parents. Children were classified into the ADHD group if the clinician's interviews and observations met DSM-5 criteria, and the parent questionnaire scores were above the threshold. If the clinician's interviews or behavioral observations did not meet DSM-5 criteria, the child was categorized into the ADHD symptom-like group. Thus, based on both age and diagnosis, there were a total of four categories of children. The study was conducted in accordance with the Declaration of Helsinki, and approved by the Medical Research Ethics Committee of the authors' institution.

### Clinical and neuropsychological assessments

All patients were interviewed by one or two experienced developmental behavior pediatricians. Overall developmental levels were assessed by the Children Neuropsychological and Behavior Scale. Regarding the evaluation of ADHD symptoms, parents of the 4-year-old group completed the Conners' Parent Symptom Questionnaire, while parents of the 5-year-old group completed the Vanderbilt ADHD Diagnostic Parent Rating Scale.

The Children Neuropsychological and Behavior Scale (CNBS) is a diagnostic assessment tool which is widely used in Chinese hospitals to assess the DQ of children aged 0–6 years (18, 19). It includes five separate subscales: gross motor, fine motor, adaptive behavior, language, and personal-social. DQ is calculated by the following formula: DQ = (mental age/chronological age) × 100. Children were tested one-on-one by a trained nurse, which takes about 20 min. Full DQ refers to the average value of the five subscales. For each subscale, a DQ less than 70 indicates a developmental delay, a DQ between 70 and 79 is slightly below the threshold for developmental delay, and a DQ of 80 or above is considered to be within the normal range (18).

The Conners' Parent Symptom Questionnaire (PSQ) is a 48-item parent-report assessment for children aged 3–17 years (20). The PSQ contains 6 subscales: conduct problem, learning problem, psychosomatic problem, impulsivity-hyperactivity, anxiety, and hyperactivity index. Symptom items are rated using a 4-point Likert scale (*never* to *very often*). Fan and colleagues developed the Chinese urban norms, which has been widely used in Chinese hospitals (21).

The Vanderbilt ADHD Diagnostic Parent Rating Scale (VADPRS) is a 55-item parent-report assessment for children aged 5–12 years (22). It contains six dimensions of symptoms: inattention (9 items), hyperactivity (9 items), oppositional defiant disorder (8 items), conduct disorder (14 items), anxiety/depression (7 items) and functional impairment (8 items). Symptom items are also rated using a 4-point Likert scale (*never* to *very often*). The VADPRS score is calculated for each subscale as sum score of parent ratings (23).

### Statistical analyses

To address the main research problem, the following analyses were conducted:

(a) Descriptive statistics were utilized to compute the mean and standard deviation of all measures.
(b) To explore the motor developmental delays among children with primary ADHD symptoms. A DQ below 70 was used as the criterion for developmental delay, while a DQ of 70 or above indicated mild or no delay (24). The rates of delayed gross motor DQ and fine motor DQ were calculated across the four categories of children.
(c) To explore the relationship between ADHD symptoms and motor development, independent sample *t*-tests were employed to compare differences in various indicators between ADHD group and ADHD symptom-like group for each age group. Pearson's correlation coefficients were calculated among all measures to explore potential correlations between different domains of DQ and ADHD symptoms. Hierarchical regression was used to examine the relationship between symptom severity and motor DQ for each group, with age, gender, adaptive behavior DQ, language DQ, and personal-social DQ as control variables.

For the 4-year-old group, symptom severity was determined by the sum of scores on the impulsive-hyperactive dimension and the hyperactivity index dimension in the PSQ. For the 5-year-old group, the severity of attention-deficit/hyperactivity symptoms was assessed by the sum of scores on the attention-deficit symptoms dimension and the hyperactive-impulsive symptoms dimension in the VADPRS.

## Results

Demographic characteristics of all the groups were shown in Table 1, including age, gender, birth weight, gestational age and birth delivery mode. The study involved 137 participants in the 4-year-old group, with 45 in ADHD group 1 (mean age 4.53 ± 0.26, 31 boys) and 92 in symptom-like group 1 (mean age 4.54 ± 0.28, 81 boys). After excluding missing values, there were 4 participants (10.81%) in ADHD Group 1 and 6 participants (8.96%) in Symptom-Like Group 1 with a gestational age of less than 37 weeks. In the 5-year-old group, there were 252 participants, with 120 in ADHD group 2 (mean age 5.47 ± 0.29, 94 boys) and 132 in symptom-like group 2 (mean age 5.50 ± 0.28, 90 boys). After excluding missing values, there were 7 participants (7.87%) in ADHD Group 2 and 5 participants (5.56%) in Symptom-Like Group 2 with a gestational age of less than 37 weeks.

**Table 1:** Demographic characteristics of different groups.

Category	Item	4-year-old group (*N* = 137)		5-year-old group (*N* = 253)	
		ADHD group 1 (*N* = 45)	Symptom-like group 1 (*N* = 92)	ADHD group 2 (*N* = 120)	Symptom-like group 2 (*N* = 132)
Child's age (in years)		4.53 ± 0.26	4.54 ± 0.28	5.47 ± 0.29	5.50 ± 0.28
Child's gender (*N*)	Female	14	9	26	42
	Male	31	81	94	90
Birth weight (*N*)	Less than 2.5 kg	1	3	4	5
	2.5 kg–4.0 kg	34	60	78	79
	More than 4.0 kg	2	4	3	4
	Missing data	8	25	35	44
Gestational age (*N*)	Less than 37 weeks (preterm)	4	6	7	5
	37–42 weeks (full term)	33	61	82	85
	Missing value	8	25	31	42
Birth delivery mode (*N*)	Vaginal delivery	17	25	48	44
	Cesarean delivery	8	27	21	23
	Missing data	20	40	51	65

Table 2 presents scores on various indicators of the CNBS and PSQ for the 4-year-old group. Both the ADHD gourp1 and symptom-like group 1 demonstrated normal gross motor development, with gross motor delay rates of 2.2% and 0%, respectively (see Table 3). However, both groups exhibited significant delays in fine motor development. The average fine motor DQ were 81.34 and 79.80, respectively, with fine motor delay rates of 20.0% and 30.4%, respectively (see Table 3). Table 4 presents scores on various indicators of the CNBS and VADPRS for the 5-year-old group. Although *t*-tests did not reveal significant differences between the two groups on CNBS scores, the symptom-like group 2 had higher rates of gross motor delay (7.6%) and fine motor delay (13.6%) compared to ADHD group 2 (6.7% and 8.3%, respectively). Similar to the 4-year-old group, *t*-tests showed that ADHD group 2 exhibited more severe oppositional defiant disorder, conduct disorder, and anxiety/depression symptoms (see Table 2).

**Table 2:** The score of the 4-year-old group on CNBS and PSQ.

Scales	No.	Index	ADHD group 1		Symptom-like group 1			
			*N* = 45		*N* = 92			
			Mean	SD	Mean	SD	*T*	*p*
CNBS	1	Full DQ	90.93	8.49	91.25	7.50	−0.22	0.825
	2	Gross motor DQ	90.47	10.38	88.53	9.84	1.07	0.289
	3	Fine motor DQ	81.34	13.02	79.80	14.85	0.59	0.554
	4	Adaptive behavior DQ	92.92	10.78	93.52	9.53	−0.33	0.739
	5	Language DQ	95.53	14.31	96.82	11.40	−0.57	0.568
	6	Personal-social DQ	93.98	11.89	97.59	9.97	−1.87	0.064
PSQ	8	Conduct problem	1.29	0.45	0.71	0.34	8.32	<0.001
	9	Learning problem	1.86	0.51	1.18	0.50	7.36	<0.001
	10	Psychosomatic problem	0.22	0.28	0.18	0.29	0.88	0.382
	11	Impulsivity-hyperactivity	2.07	0.44	1.05	0.48	11.86	<0.001
	12	Anxiety	0.57	0.43	0.42	0.33	2.33	0.021
	13	Hyperactivity index	1.82	0.39	0.99	0.38	11.79	<0.001
	14	Symptom severity^a^	3.89	0.75	2.05	0.81	12.78	<0.001

CNBS, The Children Neuropsychological and Behavior Scale; PSQ, The Conners’ Parent Symptom Questionnaire; DQ, developmental quotient.

^a^ Symptom severity = Impulsivity-hyperactivity + Hyperactivity index.

**Table 3:** Number of children with a motor developmental delay according to the subscale DQ of the CNBS (*N*, %).

Group	Gross motor		Fine motor	
	Delay (DQ < 70)	Mild to normal (DQ ≥ 70)	Delay (DQ < 70)	Mild to normal (DQ ≥ 70)
ADHD group 1	1 (2.2%)	44 (97.8%)	9 (20.0%)	36 (80.0%)
Symptom-like group 1	0 (0%)	92 (100%)	28 (30.4%)	64 (69.6%)
ADHD group 2	8 (6.7%)	112 (93.3%)	10 (8.3%)	110 (91.7%)
Symptom-like group 2	10 (7.6%)	122 (92.4%)	18 (13.6%)	114 (86.4%)

CNBS, The Children Neuropsychological and Behavior Scale; DQ, developmental quotient.

**Table 4:** The score of the 5-year-old group on CNBS and VADPRS.

Scales	No.	Index	ADHD group 2		Symptom-like group 2			
			*N* = 120		*N* = 132			
			Mean	SD	Mean	SD	*T*	*p*
CNBS	1	Full DQ	90.57	8.37	90.06	8.20	0.48	0.629
	2	Gross motor DQ	90.83	14.41	89.91	13.61	0.52	0.602
	3	Fine motor DQ	84.62	10.35	82.97	11.39	1.20	0.233
	4	Adaptive behavior DQ	89.07	12.18	89.70	10.53	−0.44	0.662
	5	Language DQ	95.81	11.38	95.47	9.10	0.26	0.793
	6	Personal-social DQ	92.46	9.14	92.31	8.96	0.14	0.892
VADPRS	15	Inattentive	16.33	3.65	10.73	3.21	12.96	<0.001
	16	Hyperactive	17.82	3.80	10.36	3.65	15.91	<0.001
	17	Oppositional defiant	10.22	4.61	7.16	3.50	5.97	<0.001
	18	Conduct disorder	3.01	2.50	1.52	1.62	5.66	<0.001
	19	Anxiety/depression	4.67	3.18	3.39	2.73	3.42	0.001
	20	Symptom severity^a^	34.15	5.42	21.09	5.95	18.16	<0.001

CNBS, The Children Neuropsychological and Behavior Scale; VADPRS, The Vanderbilt ADHD Diagnostic Parent Rating Scale; DQ, developmental quotient.

^a^ Symptom severity = Inattentive + Hyperactive.

**Figure 1:**
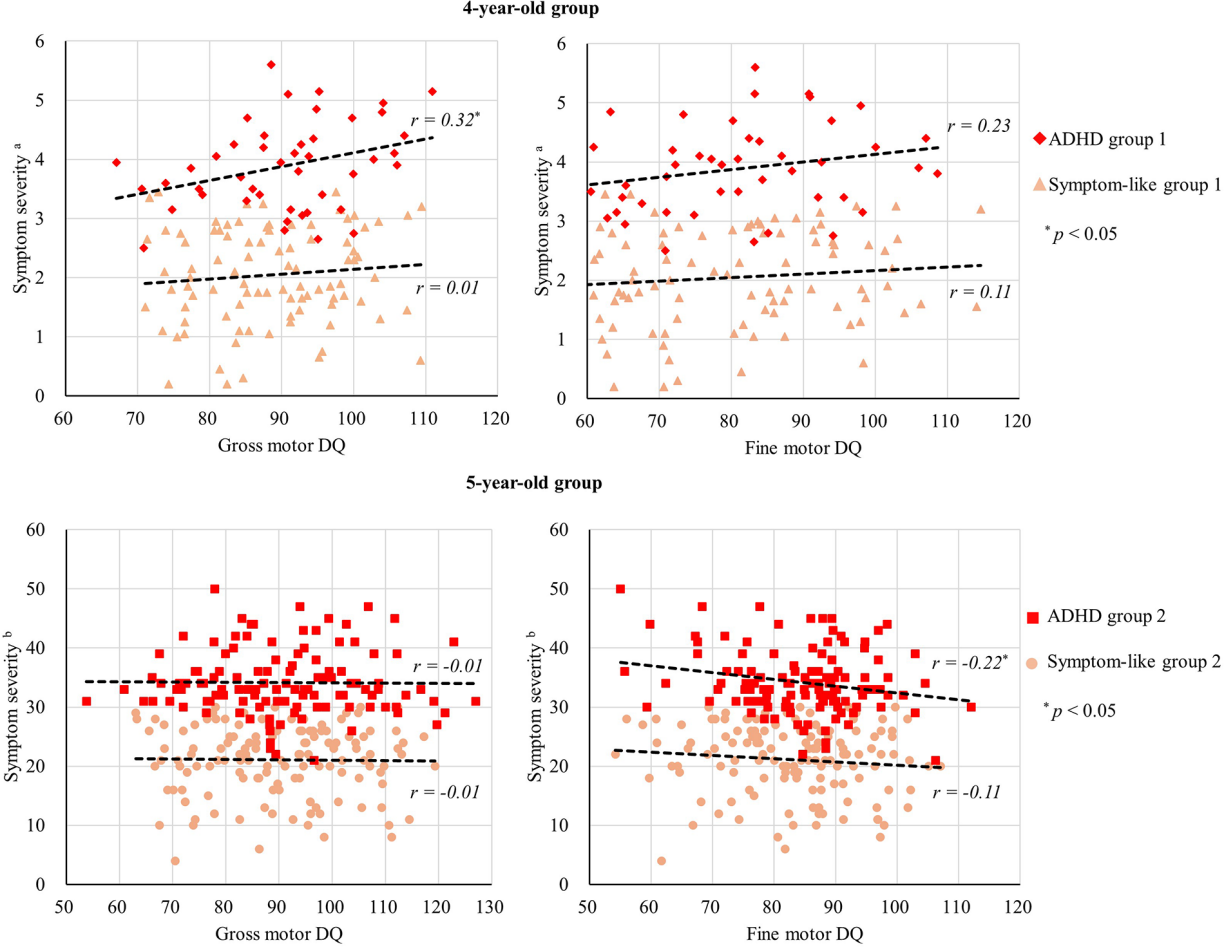
Scatter plots of gross motor DQ, fine motor DQ and symptom severity. ^a^Symptom severity = Impulsivity-hyperactivity + Hyperactivity index. ^b^Symptom severity = Inattentive + Hyperactive. The dashed line represents the regression line depicting the relationship between the *x*-axis and *y*-axis variables for each group.

Pearson correlation analysis indicated a weak correlation between gross motor DQ and hyperactivity index (*r* = 0.35, *p* = 0.020), as well as symptom severity (*r* = 0.32, *p* = 0.030) in ADHD group 1. However, fine motor DQ showed no correlation with hyperactivity index, impulsivity, or overall symptom severity in 4-year-old group (see Figure 1). Pearson correlation analysis also revealed a weak correlation between fine motor DQ and hyperactive-impulsive symptoms (*r* = −0.25, *p* = 0.006), as well as symptom severity (*r* = −0.22, *p* = 0.016) in ADHD group 2. However, gross motor DQ showed no correlation with hyperactive-impulsive symptoms, attention deficit symptoms, or overall symptom severity in 5-year-old group (see Figure 1).

Due to the four comparisons made within each age group during the hierarchical regression analysis, the alpha value was adjusted to 0.05/4 ≈ 0.017, corrected with Bonferroni correction. The hierarchical regression results indicated that, after including control variables (age, gender, adaptive behavior DQ, language DQ, and personal-social DQ), gross motor DQ in ADHD group 1 remained positively correlated with symptom severity (*β* = 0.46, *p* < 0.05, Bonferroni corrected; see Table 5). In ADHD group 2, fine motor DQ exhibited a negative correlation with symptom severity (*β* = −0.35, *p* < 0.05, Bonferroni corrected; see Table 6). The relationships between motor DQ and symptom severity were not significant in all other cases (*p* > 0.05, Bonferroni corrected).

**Table 5:** Hierarchical regression models to predict symptom severity from gross motor DQ and fine motor DQ in 4-year-old group.

Group	Explanatory variables	Step 1	Step 2a	Step 2b
		*β*	*β*	*β*
ADHD group 1	Age	−0.10	−0.01	−0.05
	Gender	−0.51*	−0.50*	−0.56*
	Adaptive behavior DQ	−0.11	−0.34	−0.24
	Language DQ	0.11	0.22	0.07
	Personal-social DQ	0.04	0.06	0.06
	Gross motor DQ		0.46*	
	Fine motor DQ			0.34
		*R*^2^ = 0.31	*R*^2^ = 0.48*	*R*^2^ = 0.40*
			Δ*R*^2^ = 0.17*	Δ*R*^2^ = 0.09
Group	Explanatory variables	Step 1	Step 2a	Step 2b
		*β*	*β*	*β*
Symptom-like group 1	Age	0.19	0.20	0.17
	Gender	−0.38*	−0.42*	−0.39*
	Adaptive behavior DQ	−0.07	−0.05	−0.08
	Language DQ	0.16	0.14	0.13
	Personal-social DQ	0.09	0.03	0.09
	Gross motor DQ		0.19	
	Fine motor DQ			0.08
		*R*^2^ = 0.21*	*R*^2^ = 0.24*	*R*^2^ = 0.21*
			Δ*R*^2^ = 0.03	Δ*R*^2^ = 0.01

The alpha value is set to 0.05/4 ≈ 0.017.

* *p* < 0.05, corrected with Bonferroni correction. The response variable is symptom severity (Impulsivity-hyperactivity + Hyperactivity index).

**Table 6:** Hierarchical regression models to predict symptom severity from gross motor DQ and fine motor DQ in 5-year-old group.

Group	Explanatory variables	Step 1	Step 2a	Step 2b
		*β*	*β*	*β*
ADHD Group 2	Age	−0.03	−0.02	−0.04
	Gender	0.20	0.22	0.25*
	Adaptive behavior DQ	0.08	0.10	0.13
	Language DQ	−0.09	−0.09	−0.05
	Personal-social DQ	0.08	0.10	0.15
	Gross motor DQ		−0.11	
	Fine motor DQ			−0.35*
		*R*^2^ = 0.05	*R*^2^ = 0.06	*R*^2^ = 0.15*
			Δ*R*^2^ = 0.01	Δ*R*^2^ = 0.10*
Group	Explanatory variables	Step 1	Step 2a	Step 2b
		*β*	*β*	*β*
Symptom-like Group 2	Age	−0.12	−0.12	−0.10
	Gender	−0.09	−0.09	−0.07
	Adaptive behavior DQ	−0.06	−0.05	−0.04
	Language DQ	−0.14	−0.14	−0.11
	Personal-social DQ	0.21	0.22	0.22
	Gross motor DQ		−0.04	
	Fine motor DQ			−0.11
		*R*^2^ = 0.05	*R*^2^ = 0.05	*R*^2^ = 0.06
			Δ*R*^2^ = 0.00	Δ*R*^2^ = 0.01

The alpha value is set to 0.05/4 ≈ 0.017.

* *p* < 0.05, corrected with Bonferroni correction. The response variable is symptom severity (Inattentive + Hyperactive).

## Discussion

The present study explored the relationship between motor development and ADHD symptom severity in preschool children aged 4.0–5.9 years. Consistent with Hypothesis 1, we found that preschool children identified by parents or teachers as exhibiting ADHD-related symptoms, regardless of whether they met the full diagnostic criteria, often showed delays in fine motor development. Contrary to Hypothesis 1, significant gross motor delays were only observed in the 5-year-old group.

Our findings regarding the rate of motor development delays in preschool children with ADHD are consistent with previous research. For instance, a nationwide epidemiological study in Egypt reported that among children aged 3–6, 2.2% experienced gross motor delays, and 2.8% experienced fine motor delays (25). However, in children with ADHD symptoms or at risk for ADHD, there is a noticeable difference in the prevalence of gross and fine motor delays. A Spanish epidemiological study reported that 22.6% of children aged 3–7 years who were likely to have ADHD had fine motor difficulties (26). In contrast, only 3.6% of children who were unlikely to have ADHD experienced similar issues (26). However, there was no significant difference in the prevalence of gross motor difficulties between the two groups, with both around 3.6% (26). This study similarly found that children exhibiting ADHD symptoms had a much higher risk of fine motor delays compared to gross motor delays, particularly among the younger children.

Consistent with Hypothesis 2, our study found a relationship between motor development and symptom severity in preschool children with ADHD. Specifically, only in the 4-year-old group, there was a positive correlation between better gross motor DQ and higher ADHD symptom severity. Conversely, only in the 5-year-old group, the ADHD group exhibited a negative correlation between poorer fine motor DQ and higher severity of parent-reported hyperactive-impulsive symptoms. No significant correlations were observed between gross motor DQ, fine motor DQ, and parent-reported ADHD symptoms in the ADHD symptom-like group.

Although ADHD symptoms are considered continuous, our study's results tend to support the interpretation that ADHD may involve specific brain developmental structures (27–30). The observed associations between motor development and symptoms in both age groups may stem from specific changes in brain structure or function during the developmental process in children with ADHD. For instance, neuroimaging studies have indicated impaired inhibitory mechanisms in the sensorimotor brain network and structural and functional alterations in the corpus callosum among children with ADHD (31, 32). The bilateral corticospinal tract (CST), which transmits motor signals from the primary motor cortex down the spinal cord to the trunk and limb muscles, may also serve as a crucial brain structure linking motor skills and ADHD symptom severity. Previous study on children aged 9–11 has found that increased ADHD symptom severity is associated with reduced white matter organization in fronto-pontine fibers projecting to and from the supplementary motor area (14). Furthermore, increased fiber density in the CST during adolescence and early adulthood has been linked to a greater reduction in hyperactivity and impulsivity symptoms over the preceding 3–4 years (17).

Furthermore, the inconsistency in the motor-symptom associations between fine and gross motor skills across different age groups may arise from differences in the developmental rates of these two types of movements, leading to atypical development of motor-related brain networks. The negative correlation between fine motor skills and symptom severity, consistent with previous findings in school-aged children, suggests that fine motor impairments correlate with ADHD symptoms (33). However, the positive correlation between gross motor skills and symptom severity indicates that this atypical motor development cannot solely be attributed to fine motor impairment. Unlike in school-aged children (34), the results of this study suggest that, for younger children, higher levels of gross motor development may not provide a protective effect against ADHD symptoms.

The present study may inform future interventions targeting motor skills in preschool children exhibiting ADHD symptoms. For younger children, it is important to ensure they have sufficient opportunities for gross motor development while simultaneously encouraging participation in fine motor activities, such as using chopsticks or buttoning clothing. For older preschoolers, a comprehensive approach should be taken to monitor both gross and fine motor development. Activities such as ball games, climbing, stringing beads, and playing with playdough can be beneficial for the development of either gross or fine motor skills. Previous research on school-aged children has also suggested that motor interventions can reduce ADHD symptoms and enhance cognitive and academic outcomes (3, 10, 35, 36). When designing interventions to address the motor abilities of children with ADHD, it is vital to consider their developmental stage and the specific processes affecting the corresponding motor-related brain networks.

### Limitations and strengths

Interpretation of the results should be tempered by the limitations of this study. The sample was drawn from a clinic population of Chinese preschool children with reported attention problems, lacking healthy controls. The existing data derived from the clinic-based sample may introduce bias and may not accurately represent the broader preschool population. Furthermore, the utilization of different ADHD parent questionnaires for the two preschool groups complicates the comparison of ADHD symptom severity between them and may impact result interpretation.

Despite these limitations, the study possesses several strengths. It filled a gap in previous research concerning the relationship between fine motor development and ADHD in preschool children. By considering the continuum of ADHD symptoms and investigating both children meeting diagnostic criteria and those with symptomatic presentations, the study results are more generalizable. By distinguishing between fine and gross motor development, this study offers insights for future motor intervention strategies targeting preschool children with ADHD.

As a retrospective cross-sectional study, the present study offers preliminary insights into the relationship between ADHD symptoms and motor DQ in children across two age groups. However, we acknowledge that further longitudinal studies would be valuable in exploring the developmental trajectories of motor skills in children with ADHD symptoms. Additionally, exploring the brain structures and functions of motor-related brain regions will also enhance our understanding of this research field.

## Conclusion

The results of present study indicate that preschool children with ADHD symptoms exhibit high rates of motor developmental delays, particularly in fine motor development, regardless of whether they meet diagnostic criteria. However, whether in the 4-year-old group or the 5-year-old group, correlations between ADHD symptom severity and motor development were found only within the ADHD group. Therefore, the study indicates a need for increased focus from both parents and healthcare professionals on the motor development status of children meeting diagnostic criteria for ADHD.

## Data Availability

The datasets presented in this article are not readily available because these medical data involve the privacy of the child. Requests to access the datasets should be directed to Aimin Liang, liang-aimin@163.com.
